# Fate of Benzalkonium Chloride in Nanofiltration and Reverse Osmosis: Mechanisms of Retention and Membrane Response

**DOI:** 10.3390/molecules31091532

**Published:** 2026-05-05

**Authors:** Aleksandra Klimonda, Gabriela Kamińska, Izabela Kowalska, Krzysztof Barbusiński

**Affiliations:** 1 Faculty of Environmental Engineering, Wroclaw University of Science and Technology, Wybrzeże S. Wyspiańskiego 27, 50-370 Wrocław, Poland; aleksandra.klimonda@pwr.edu.pl (A.K.); izabela.kowalska@pwr.edu.pl (I.K.); 2 Department of Water and Wastewater Engineering, Silesian University of Technology, ul. Akademicka 2A, 44-100 Gliwice, Poland; krzysztof.barbusinski@polsl.pl

**Keywords:** quaternary ammonium compounds, pressure-driven membrane process, cationic surfactants, zeta potential, MWCO, Hermia model, disinfectants, emerging contaminants, micropollutants, persistent and mobile chemicals

## Abstract

Cationic surfactants from quaternary ammonium compounds (QACs) are increasingly recognized as relevant micropollutants particularly following their widespread use during and after the COVID-19 pandemic. The new EU Urban Wastewater Treatment Directive (2024/3019) highlights micropollutant removal as a regulatory priority, mandating advanced treatment for their elimination. In this context, this study examined benzalkonium chloride (BAC) retention and membrane response during nanofiltration (NF) and reverse osmosis (RO), across concentrations ranging from monomeric to micellar. RO membranes achieved >97% rejection, whereas NF showed 65–96% removal strongly affected by micelle formation. Flux decline was most pronounced in RO, with relative permeability (*J*/*J*_0_) decreasing to ~0.12 at 1.0 CMC, while NF membranes exhibited better hydraulic stability. Membrane active layer zeta potential measurements confirmed adsorption and charge neutralization, with shifts toward less negative values after BAC exposure. Hermia model analysis revealed that fouling was governed by cake layer formation or pore blocking, depending on membrane type and feed concentration.

## 1. Introduction

Pressure-driven membrane processes (PDMPs), including nanofiltration (NF) and reverse osmosis (RO), represent established technologies in advanced water and wastewater treatment. Beyond nominal MWCO, membrane efficiency is strongly influenced by selective-layer properties such as hydrophilicity and zeta potential, which dictate the balance between size exclusion and solute–surface interactions, and thus determine separation and fouling behaviour [1,2,3,4].

To optimize membrane performance, various surface modifications have been introduced either during fabrication or by post-treatment of commercial membranes [5]. Within this broad spectrum, surfactants have emerged as simple and cost-effective modifiers capable of improving hydrophilicity, altering surface charge, or imparting antimicrobial activity. For instance, Fang et al. [6] showed that adding SDS surfactant during polyamide membrane fabrication enhanced its wettability, with the contact angle decreasing from ~80° (no surfactant) to ~60° at 0.4 CMC and to ~40° at 1.0 CMC. Chew et al. [7] demonstrated that the contact angle of PVDF membrane decreased from 116° to 64° after exposure to the non-ionic surfactant Tween 20, and to 91° after exposure to the anionic surfactant SDS. Khan et al. [8] reported that surfactant-modified ceramic membranes achieved up to 98.5% oil–water separation and exhibited more stable flux, compared to limited performance without surfactants. Beyond improving surface wettability, surfactants have also been employed to impart antimicrobial functionality. For example, Cihanoğlu and Altinkaya [9] prepared PES UF membranes with CTAB that showed strong antibacterial activity on both Gram-negative (*Escherichia coli*) and Gram-positive (*Staphylococcus aureus*) bacteria.

The same interfacial activity that makes surfactants useful as modifiers can also impair membrane performance during wastewater treatment through adsorption, micelle deposition, and charge neutralization. This challenge is particularly pronounced for cationic surfactants from the quaternary ammonium compound (QAC) group, with benzalkonium chloride (BAC) as a widely used representative produced at over 700,000 tons annually [10]. The issue has been further exacerbated by the sharp increase in disinfectant formulations following the COVID-19 pandemic [11,12].

Quaternary ammonium surfactants, including BAC, have recently been recognized as part of the broader group of persistent and mobile (PM) chemicals, an emerging issue of concern for both environmental and human health [13]. In parallel, the recently adopted EU Urban Wastewater Treatment Directive (2024/3019) requires large treatment plants to implement advanced treatment for micropollutant removal while revisions to the Water Framework Directive broaden the list of priority substances [14]. Although cationic surfactants such as BAC are not named explicitly, their widespread application in disinfectants, detergents, and personal care products places them within the scope of these regulatory developments [15].

Pressure-driven membrane processes (PDMPs) are increasingly applied for surfactant removal, though their efficiency varies with membrane type and solute concentration. The ability of ultrafiltration (UF) to remove surfactants is limited to concentrations exceeding the critical micelle concentration (CMC), whereas at lower concentrations, dense NF and RO membranes provide more effective separation [16,17]. Membrane studies have shown that low-pressure membranes provide limited surfactant removal. Tomczak and Gryta [18] observed 28% and 15% removal of SDS (130 mg/L, ~0.05 CMC) using PES UF membranes with 10 and 100 kDa MWCO, respectively. Kim and Park [19] tested ceramic UF membranes for SDBS (CMC ~ 450 mg/L) solutions at initial concentrations of 100, 500, and 1000 mg/L. While membranes with MWCO of 15 and 150 kDa achieved less than 40% removal, only the dense 1 and 5 kDa membranes provided appreciable rejection, up to 50–70%.

Given the limited efficiency of UF, particularly at sub-CMCs, more compact membranes are required. NF and RO offer substantially higher rejection and are therefore more suitable for surfactant removal. Badeuquin et al. [20] reported 99.4% removal of fluorinated surfactants in RO process. According to Halleb et al. [21], PA and CA RO membranes consistently removed over 90% of three types of surfactants (anionic, cationic, non-ionic) across a concentration range of 25–10,000 mg/L, based on TOC measurements.

Among the three main surfactant classes, cationic surfactants appear to exhibit the highest fouling propensity, causing a more severe permeate flux decline than nonionic and anionic surfactants. In particular, Ishida et al. [22] demonstrated that CTAB caused the strongest permeability loss in polyamide RO membranes, reducing flux by approximately 65%, compared with about 45% for Triton X-100 (non-ionic) and 30% for SDS (anionic). This was attributed to the strong electrostatic attraction between the positively charged surfactant head group and the negatively charged membrane surface, which promotes adsorption. In addition, the outward-oriented hydrophobic tails may increase the apparent membrane hydrophobicity, thereby further enhancing fouling [23].

Although pressure-driven membrane processes are promising for surfactant removal, mechanistic evidence for cationic quaternary ammonium compounds remains limited. Many membrane studies have focused on cetyltrimethylammonium bromide (CTAB) as a single-chain model surfactant [21,22,23,24], whereas benzalkonium chloride (BAC) has been much less systematically investigated in comparative NF/RO systems despite its greater environmental relevance. Unlike CTAB, BAC is not a single compound but a homologous mixture of alkylbenzyldimethylammonium chlorides with different alkyl chain lengths, and this compositional heterogeneity may affect micellization, membrane–solute interactions, retention, and fouling behaviour, especially across the transition from monomeric to micellar form. Previous work by Klimonda and Kowalska [25] demonstrated the applicability of polymeric membranes for the purification of solutions containing cationic surfactants, but did not provide a BAC-focused mechanistic comparison across multiple NF and RO membranes. Therefore, the present study systematically evaluates BAC removal by commercial NF and RO membranes over the concentration range from 0.1 to 1.0 CMC and relates separation performance to flux decline, zeta potential shifts, and Hermia model fouling analysis, thereby providing a more complete mechanistic description of BAC behaviour in pressure-driven membrane processes.

## 2. Results and Discussion

### 2.1. Separation Properties

**Figure 1:**
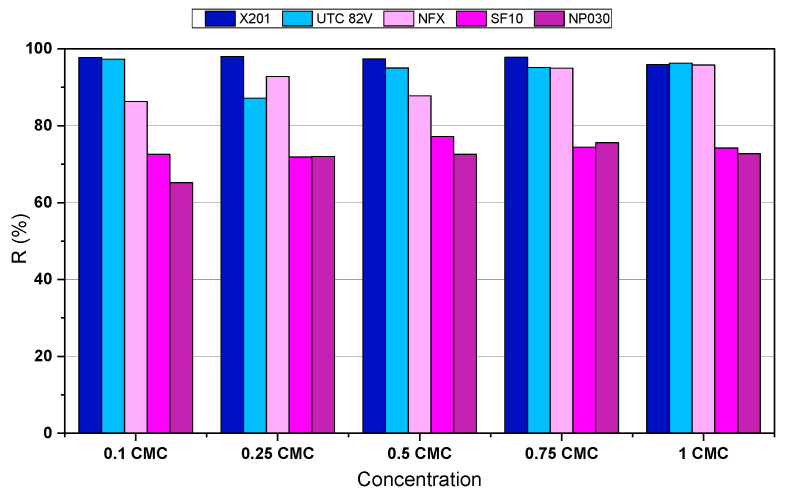
BAC retention (R,%) efficiency on X201, UTC-82V, NFX, SF10, and NP030 membranes (TMP = 20 bar, T = 22 °C).

The effectiveness of the membranes was evaluated in terms of BAC retention (Figure 1). Rejection closely followed the nominal pore size, with denser structures providing higher removal. The RO membrane X201 exhibited excellent performance, maintaining >95% retention across the entire concentration range. The UTC-82V membrane performed similarly, although a slight decline was noted at 0.25 CMC (87%). In contrast, NF membranes with more open structures showed reduced efficiency. The polyamide-based NFX (MWCO 150–300 Da) provided the highest NF retention, increasing from 86.3% at 0.1 CMC to 95.8% at 1.0 CMC, indicating a clear contribution of micelle formation. The SF10 (300–500 Da) achieved only moderate rejection (71.8–77.2%), while the NP030 (500–600 Da) was the least effective, with 65.2–72.7% retention. A comparable concentration-dependent trend was reported by Kim and Park [19] for the anionic surfactant SDBS (100–1000 mg/L): tight UF membranes (1–5 kDa) achieved markedly higher rejection, whereas looser UF (10–300 kDa) remained ineffective.

As BAC concentration increased, the solute progressively shifted from predominantly monomeric to aggregated form. For quantitative comparison with membrane selectivity, BAC monomers are more appropriately described by their molecular-weight range than by a geometric length estimate. Depending on alkyl chain length, BAC monomers fall within approximately 283.8–423.97 Da, which enables direct comparison with the nominal MWCO of the NF membranes used in this study: ~150–300 Da for NFX, 300–500 Da for SF10, and 500–600 Da for NP030. This relationship helps explain the observed NF rejection trend, in which NFX showed the highest removal, while the more open SF10 and NP030 membranes were more permissive to BAC transport at sub-CMC conditions.

With increasing concentration, BAC micellization became increasingly important. The measured aggregate diameter was 11.8 ± 1.0 nm, and, considering the reported aggregation number of 10–60 [26,27], the corresponding apparent aggregate molecular mass is on the order of several to tens of kDa. These aggregates therefore greatly exceed the MWCO range of the NF membranes, which explains the increase in rejection with concentration, especially for the tight NFX membrane. This, together with the mean pore diameter of NF membranes typically reported to be below 0.8 nm [28,29], and even smaller effective pores in RO membranes [30], may explain the higher rejection observed in PDMPs compared to monomeric solutions.

### 2.2. Transport Properties

**Figure 2:**
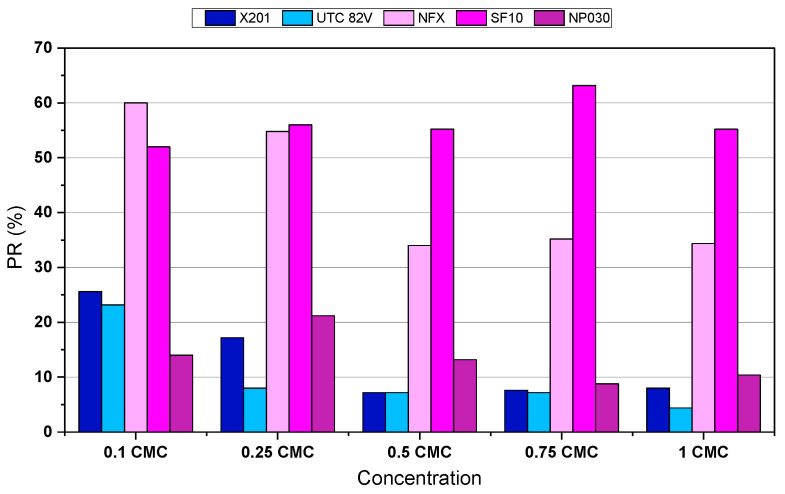
Permeate recovery (PR,%) of X201, UTC-82V, NFX, SF10 and NP030 membranes (TMP = 20 bar, T = 22 °C) during the 120 min membrane process.

The permeate recovery ratio after 120 min (Figure 2) shows a strong relationship between membrane pore size and permeability. In general, membranes with larger nominal pore sizes demonstrated higher recovery, whereas tighter membranes yielded considerably lower recovery values, particularly at higher BAC concentrations. However, despite its relatively high porosity (MWCO ~500–600 Da), the NP030 membrane achieved only 8.8–21.2% recovery, likely due to its lower hydrophilicity (active layer made of PES material) and possible fouling interactions with BAC monomers or aggregates.

**Figure 3:**
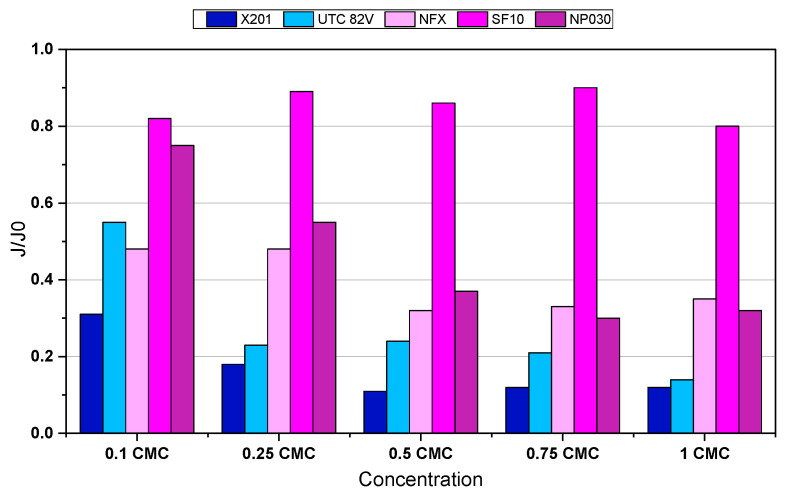
Normalized flux (*J*/*J*_0_) of X201, UTC-82V, NFX, SF10, and NP030 membranes (TMP = 20 bar, T = 22 °C) during the 120 min membrane process.

To complement the analysis of permeate recovery and better understand the fouling behaviour, changes in membrane permeability were further examined through normalized flux (*J*/*J*_0_) measurements after 120 min of filtration (Figure 3). A decline in membrane permeability over the 120 min filtration cycle was observed for most membranes, with the effect particularly pronounced for RO membranes, indicating severe flow resistance caused by fouling, whereas NF membranes exhibited a more moderate decline.

Among the tested membranes, SF10 exhibited the highest operational stability. Its *J*/*J*_0_ values remained between 0.80 and 0.90, even at higher BAC concentrations, indicating low fouling susceptibility. This behaviour appears to be associated mainly with its favourable surface properties (highly hydrophilic active layer), which may have compensated for the moderately open pore structure and reduced micelle adhesion and pore blocking. The present results are in agreement with the observations reported by Ishida et al. [22], who investigated surfactant fouling of polymeric membranes and found that surfactants had only a minor effect on the water flux of cellulose acetate membranes.

In contrast, the RO membranes X201 and UTC-82V exhibited the most significant decline in hydraulic performance. At the highest concentration (1.0 CMC), their normalized flux amounted to 0.12 and 0.14, respectively, confirming strong susceptibility to fouling and concentration polarization effects. These membranes, while highly selective, are more prone to surface accumulation of micelles, which likely formed a dense adsorption layer impeding water transport. The severe flux decline observed for the RO membranes can be attributed to the combined effects of high BAC rejection, concentration polarization, and strong membrane–surfactant interactions at the membrane surface. Because X201 and UTC-82V retained BAC very effectively, surfactant molecules were likely enriched in the near-membrane region during filtration. Under such conditions, local BAC concentration at the membrane interface may approach or exceed the aggregation threshold, promoting the accumulation of monomers and micellar structures in the form of a dense adsorption/polarization layer. In addition, the negative surface charge of the RO membranes in the experimental pH range favoured electrostatic attraction toward the permanently charged cationic BAC headgroup, which further supported surface deposition. As a result, the hydraulic resistance increased substantially, leading to a pronounced decline in relative flux. The observed changes in zeta potential and their relation to BAC adsorption and membrane performance are discussed in detail in Section 2.3.

**Figure 4:**
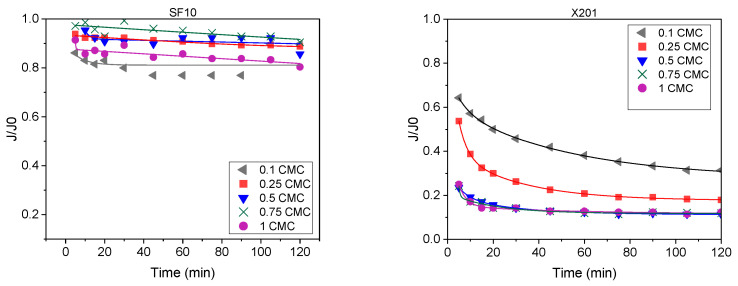
Normalized flux (*J*/*J*_0_) during 120 min filtration for the NF membrane SF10 and the RO membrane X201 at different BAC concentrations (TMP = 20 bar, T = 22 °C).

To complement the endpoint permeability values, flux evolution over time was analyzed for two representative membranes with contrasting fouling behaviour (Figure 4). The SF10 membrane maintained *J*/*J*_0_ between 0.80 and 0.90 throughout the 120 min cycle, indicating limited fouling, whereas the RO membrane X201 showed a rapid decline below 0.2 at 0.5, 0.75 and 1.0 CMC. The time-course plots of normalized flux (*J*/*J*_0_) for all membrane/concentration systems are provided in the Supplementary Materials.

It should be noted that the present study was conducted using model BAC solutions prepared in distilled water in order to isolate the effect of surfactant concentration. In real wastewater, coexisting salts, natural organic matter, and other solutes may modify BAC aggregation, electrostatic interactions, and fouling behaviour. Ionic strength is known to shift the micellization process of ionic surfactants, typically lowering the CMC by screening electrostatic repulsion between charged head groups [31], while water-matrix constituents can also alter solute transport and membrane rejection in NF/RO systems [32]. Moreover, NOM and multivalent ions may promote additional organic fouling and change membrane–solute interactions [33]. Therefore, the retention and flux decline observed under real conditions may differ from those obtained for single-component systems.

### 2.3. Membrane Surface Characteristics

**Figure 5:**
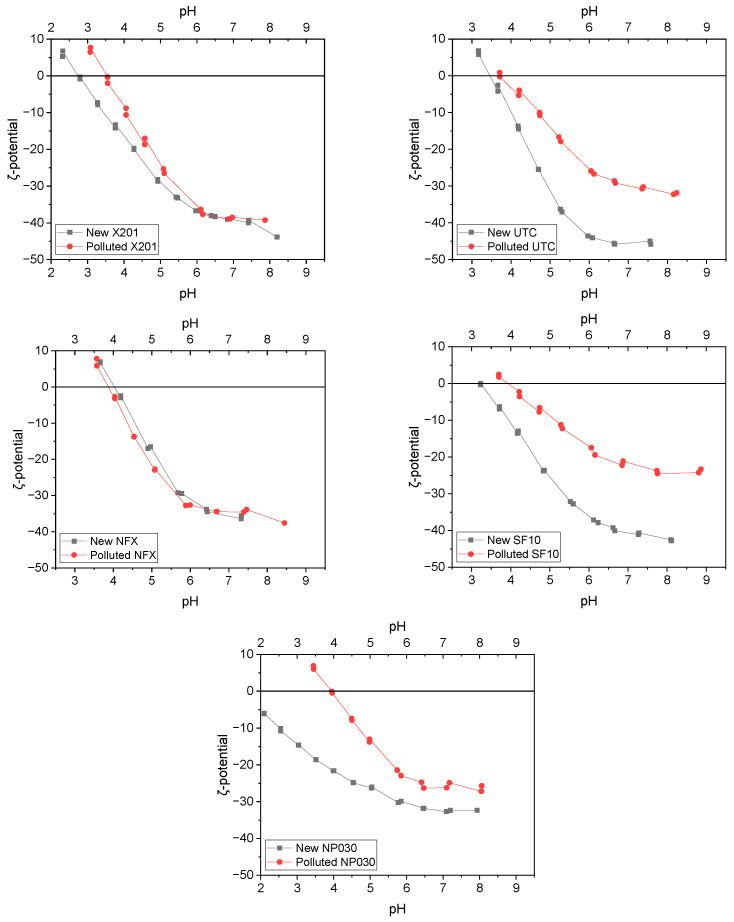
Zeta potential [mV] as a function of pH for the X201, UTC-82V, NFX, SF10 and NP030 membrane before and after 120 min filtration of 0.5 CMC BAC solution.

Given the observed fouling tendencies during BAC filtration, zeta potential measurements were carried out to assess the surface charge properties of the membranes, which play a key role in electrostatic interactions during PDMPs. As shown in Figure 5, new membranes showed isoelectric points (pI) between 2.5 and 4.1, being positively charged at lower pH and negatively charged above their respective pI values. After BAC filtration, both the zeta potential curves and isoelectric points shifted toward higher values, indicating changes in the membrane surface chemistry that likely contributed to fouling. Only the NFX membrane maintained an almost identical zeta profile before and after exposure, suggesting limited interaction with BAC.

Notably, the pH of BAC solutions used during filtration experiments was in the range 7–8, making this range particularly relevant for interpreting electrostatic interactions and fouling tendencies. BAC remains positively charged across the studied pH range due to its permanent cationic headgroup. As all membranes exhibited negative zeta potential between pH 7–8, electrostatic attraction likely contributed to BAC adsorption and subsequent fouling.

All the membranes tested exhibited negative surface charge at pH range 7–8; however, the UTC membrane exhibited the most negative zeta potential (−45 mV at pH 7–8), which likely promoted strong electrostatic attraction with cationic BAC macromolecules, leading to extensive adsorption and fouling, as reflected by low normalized flux (*J*/*J*_0_) values (Figure 3). In contrast, the SF10 membrane, with a less negative surface charge (~−33 mV), showed a comparatively higher *J*/*J*_0_, suggesting reduced BAC accumulation. This indicates that surface charge intensity plays a role in fouling behaviour during PDMPs of cationic surfactants. This interpretation is consistent with previous reports showing that oppositely charged membrane–surfactant systems, such as negatively charged membranes in contact with cationic surfactants, are particularly prone to pronounced flux decline [34].

Following 120 min filtration of 0.5 CMC BAC, the zeta potential curves of X201, UTC-82V, SF10 and NP030 shifted toward less negative values, whereas NFX remained nearly unchanged (Figure 5). This behaviour indicates that BAC species remained associated with the membrane surface during electrokinetic measurement, which is consistent with partial charge masking and neutralization of the initially negative active layer by the permanently cationic surfactant. However, the present results do not allow a rigorous quantitative separation between reversible accumulation within the concentration-polarization/cake layer and irreversible adsorption to the active layer, because no dedicated rinsing, cleaning, desorption, or flux-recovery experiments were performed. Therefore, the zeta-potential shift should be interpreted as evidence of persistent post-exposure surface modification rather than as direct proof that the entire foulant layer was irreversibly bound.

The relationship between surface charge change and filtration performance can be further explained by the surface-sensitive nature of zeta potential. At pH 7–8, all tested membranes were negatively charged, while BAC remained positively charged, favouring electrostatic attraction and local BAC accumulation. This effect was particularly pronounced for UTC-82V, which exhibited the most negative zeta potential and severe flux decline, whereas SF10 showed a less negative surface and much higher normalized flux. At the same time, the results indicate that surface charge was not the only controlling factor, since membrane compactness, hydrophilicity, and surface-layer growth also contributed to the final filtration performance.

Beyond the observed shift in zeta potential, BAC deposition may also influence other membrane surface properties. Adsorbed BAC molecules and/or micellar aggregates may create a surface layer that alters the apparent hydrophilicity and effective roughness of the membrane. Comparable behaviour has been reported for other cationic surfactants; Tang et al. [23] showed that exposure to CTAB increased the contact angle of polyamide RO membranes from 51.9° to 54.9° after 1 h. Such surface modifications may additionally promote fouling and increase hydraulic resistance. While these effects were not directly examined in the present study, they should be considered as a complementary mechanism accompanying electrostatic interactions.

### 2.4. Fouling Mechanisms—Hermia Model Analysis

**Figure 6:**
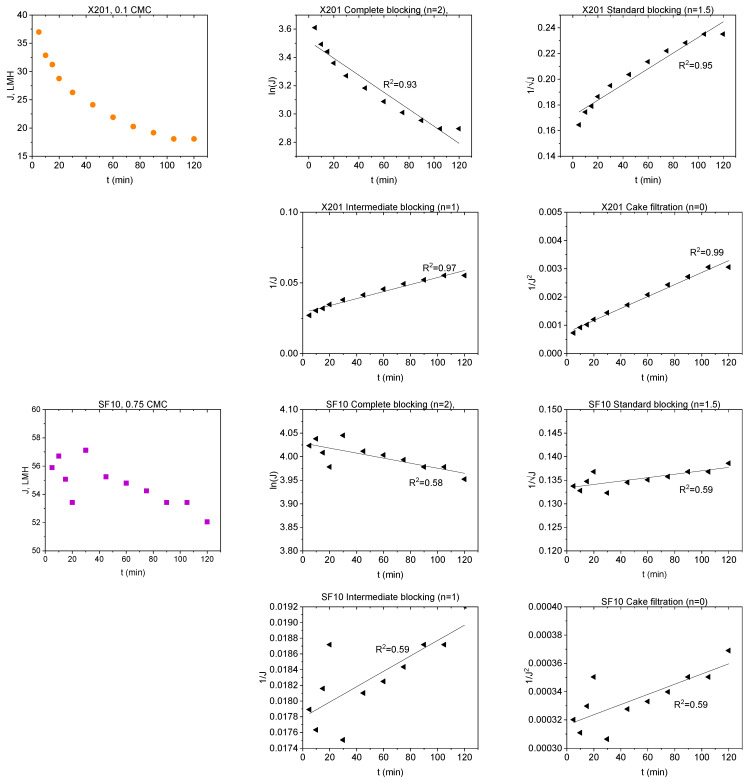
Experimental flux decline curves and corresponding linearized Hermia plots for two representative cases: X201–0.1 CMC and SF10–0.75 CMC. The Hermia analysis includes complete blocking, standard blocking, intermediate blocking, and cake filtration models; R^2^ values are given in the individual panels.

To gain further insight into the fouling behaviour during BAC filtration, the experimental flux decline data were fitted using Hermia’s classical blocking models (complete, standard, intermediate, and cake filtration) under constant-pressure conditions. Table 1 summarizes Hermia model fitting results expressed as coefficients of determination (R^2^) for the tested membrane–BAC systems. In the present study, Hermia fitting is interpreted as a phenomenological description of the dominant hydraulic resistance pattern rather than as direct proof of a single microscopic fouling event. Representative experimental flux decline curves and the corresponding linearized Hermia plots for selected NF and RO membrane cases are presented in Figure 6.

The observed differences between membranes can be explained by the combined effect of membrane compactness and BAC aggregation state. Below the CMC, BAC is present predominantly as monomers and small pre-micellar associates, whereas with increasing concentration toward the CMC the contribution of larger aggregates becomes more significant. In addition, under stirred dead-end filtration, concentration polarization may increase the local BAC concentration near the membrane surface. As a result, even when the bulk feed concentration remains below the CMC, the interfacial concentration at the membrane surface may still become sufficiently high to promote adsorption-assisted surface layer formation.

For the RO membranes (X201 and UTC-82V) and the tight NF membrane (NFX), the dense selective layer limited penetration of BAC species into the membrane interior. Consequently, rejected BAC accumulated mainly at the membrane/solution interface. Because BAC remained permanently cationic, while the membrane surfaces were negatively charged at pH 7–8, electrostatic attraction likely facilitated the initial adsorption step and promoted further surface deposition. Therefore, flux decline for these membranes was consistently best represented by the cake filtration model across the investigated concentration range. The relatively high R^2^ values obtained at 0.1–0.5 CMC indicate stable development of a BAC-rich surface layer, whereas the lower R^2^ values observed at higher concentrations likely reflect simultaneous layer growth, compression, and restructuring rather than ideal steady-state cake formation alone. This interpretation is consistent with the severe permeability loss observed for X201 and UTC-82V.

A different behaviour was observed for the more open NP030 membrane. At sub-CMC conditions, BAC monomers and small pre-micellar associates could more readily access pore structure and internal transport pathways, which explains the blocking-type behaviour identified mainly at 0.25–0.5 CMC. In contrast, the very low R^2^ obtained at 0.1 CMC indicates that no reliable mechanistic assignment can be made for that condition. As BAC concentration increased further, pore penetration became less favourable because of the increasing contribution of larger BAC associates and stronger surface accumulation. Accordingly, the dominant mechanism shifted toward cake filtration, especially at 1.0 CMC, indicating that flux decline became increasingly governed by surface layer formation rather than internal pore obstruction.

The SF10 membrane displayed the highest resistance to BAC fouling. Its normalized flux remained relatively stable throughout the 120 min experiments, which resulted in weak discrimination between Hermia models for most concentrations. Because the decrease in *J*/*J*_0_ during filtration was only weakly pronounced, the extent of permeability loss was too small to allow reliable differentiation between the linearized model forms. Only at 0.25 CMC did a meaningful cake filtration fit emerge, whereas the low R^2^ values at the remaining concentrations should be interpreted cautiously and taken mainly as confirmation of limited fouling development rather than evidence of a single well-defined mechanism. The comparatively favourable hydraulic stability of SF10 is consistent with the low flux decline observed experimentally and may be related to the hydrophilic character of its cellulose acetate active layer, which likely reduced BAC affinity and limited formation of a resistant foulant layer.

Taken together, the Hermia analysis indicates that BAC fouling was governed by both membrane structure and surfactant aggregation state. Dense RO and tight NF membranes were dominated by surface-controlled fouling from the onset of filtration, whereas the more open NP030 membrane was more susceptible to pore blocking at sub-CMC conditions before shifting toward cake formation at higher concentrations. Thus, the dominant fouling mechanism was not controlled solely by nominal MWCO, but also by the location of hydraulic resistance development and by the concentration-dependent state of BAC in solution.

## 3. Materials and Methods

### 3.1. Chemicals

Benzalkonium chloride (BAC, (≥80% purity) was used as a model cationic surfactant. BAC is a mixture of alkylbenzyldimethylammonium chlorides with varying alkyl chain lengths (typically C_12_, C_14_, and C_16_). Its general molecular formula can be represented as C_6_H_5_CH_2_N(CH_3_)_2_RCl, where R denotes a C_8_H_17_–C_18_H_37_ alkyl chain. The molecular weight of a representative BAC molecule (benzalkonium chloride C_12_) is approximately 340.0 g/mol. BAC feed solutions were freshly prepared in deionized (conductivity 2.4 µS/cm) water at concentrations corresponding to 0.1, 0.25, 0.5, 0.75, and 1.0 CMC. No buffer or background electrolyte was added, so the feed matrix represented a simplified BAC-in-water system intended to isolate BAC–membrane interactions. The initial pH of the feed solutions was 6.5 and was not adjusted.

The critical micelle concentration of BAC has been determined by the authors [25] to be 350 mg/L, with aggregate size distribution indicating micelles of 11.8 ± 1.0 nm in diameter. These parameters provided the reference framework for the concentration ranges applied in this study, with feed solutions prepared at 0.1, 0.25, 0.5, 0.75, and 1.0 times the CMC.

### 3.2. Set-Up

**Figure 7:**
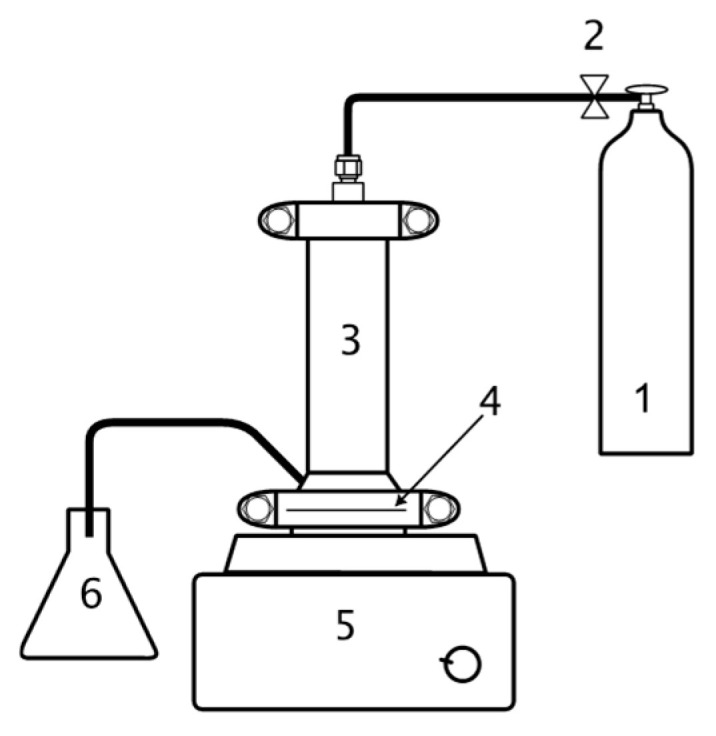
Laboratory set-up. 1—gas; 2—pressure regulator; 3—filtration cell; 4—membrane; 5—magnetic stirrer; 6—permeate collection.

All filtration experiments were performed using a Sterlitech HP4750 (Sterlitech Corporation, Auburn, WA, USA) stirred dead-end filtration cell (Figure 7). The system was operated in batch mode under constant pressure conditions. The membrane cell had an effective filtration area of 14.6 cm^2^ and was equipped with a magnetic stir bar rotating at ~300 rpm to minimize concentration polarization near the membrane surface. Compressed nitrogen gas was used to apply a constant transmembrane pressure of 20 bar throughout the experiment. The feed solution volume was 250 mL, and all experiments were carried out at ambient temperature (22 ± 1 °C). Permeate samples were collected manually at specific time intervals to determine volumetric flux.

Prior to BAC filtration, the hydraulic behaviour of each membrane type was initially characterized on a representative fresh coupon by measuring the distilled-water flux at 5, 10, 15, 20, and 30 bar. For subsequent routine experiments, each fresh membrane coupon was conditioned with distilled water at 20 bar until the pure-water flux approached the stabilized reference value previously established for that membrane type. This stabilized distilled-water flux at 20 bar was taken as *J*_0_ for flux normalization. Because the time required for stabilization varied slightly between coupons, conditioning was defined by the attainment of a near-stable flux rather than by a fixed pre-compaction time.

### 3.3. Membranes

Five commercially available flat-sheet membranes were employed, covering both reverse osmosis (RO) and nanofiltration (NF) types. These membranes differed in material composition, operating pH range, target applications and rejection performance. The selection was intended to enable a comparative analysis of their separation efficiency and resistance to fouling caused by organic micropollutants. Among the tested membranes, NP030 membrane, according to the producer information [35], exhibits nanofiltration characteristics when exposed to high pressure. Membrane NFX represents nanofiltration (MWCO ~150–300 Da). The SF10 membrane, with an intermediate MWCO of ~300–500 Da, lies at the interface between tight UF and loose NF membranes. Technical characteristics of the membranes used in the study, including recommended feed type, polymer composition, retention properties, and pH stability ranges are listed in Table 2.

### 3.4. Process Performance Assessment

To evaluate membrane retention performance, samples of the feed and permeate were analyzed for BAC concentrations. Quantification was performed using UV-Vis spectrophotometry at a wavelength of λ = 215 nm, corresponding to the absorption maximum of BAC in aqueous solutions. Measurements were carried out using a Shimadzu UV-1240 Mini spectrophotometer (Shimadzu, Kyoto, Japan) with quartz cuvettes and a 1 cm optical path length.

The BAC retention (*R*, %) was calculated as:

$$R=(1-\frac{C_{p}}{C_{0}})\cdot 100\%$$

where *C*_0_ is the initial feed concentration and *C_p_* is the BAC concentration in the permeate.

The volumetric flux (*J*, L·m^−2^·h^−1^) was calculated from the permeate volume collected over time using the equation:

$$J=\frac{V}{A\cdot t}$$

where *V* is the permeate volume (L), *A* is the effective membrane area (m^2^), and *t* is the filtration time (h).

For comparative analysis, flux values were normalized to the distilled water flux (*J*/*J*_0_, -).

Permeate recovery (*PR*, %) was calculated as the ratio of collected permeate volume to the initial feed volume:

$$PR=\frac{V_{p}}{V_{F}}\cdot 100\%$$

where *V_p_* is total volume (L) of permeate collected during the entire filtration process, *V_F_* is an initial volume of the feed solution (L).

### 3.5. Zeta Potential Analysis

Zeta potential and isoelectric point of the membranes were measured before and after BAC exposure using a SurPASS Electrokinetic Analyzer (Anton Paar GmbH, Graz, Austria). The system was equipped with an automatic titration system. Measurements were carried out with 0.01 mol/L KCl. The pH was adjusted automatically over the range [2,3,4,5,6,7,8,9] using HCl/NaOH.

### 3.6. Hermia Model Fitting

To investigate fouling mechanisms during BAC filtration, experimental permeate flux data were fitted using Hermia’s classical blocking models [36]. The principles of these models under constant-pressure conditions are summarized in Table 3.

## 4. Conclusions

This work demonstrated that the removal of benzalkonium chloride and fouling behaviour in pressure-driven membrane processes depends not only on membrane tightness but also on surface interactions and the aggregation state of the surfactant. RO membranes provided the highest rejection (>97%) but were most prone to severe flux decline, whereas NF membranes exhibited lower but concentration-dependent removal with better hydraulic stability.

The analysis indicated that loose NF/UF membranes were affected by pore blocking at sub-CMCs, while tight NF and RO membranes were consistently governed by cake filtration. The SF10 membrane remained the most resistant, showing negligible fouling. At and above the CMC, all membranes converged toward cake formation, though with different stability.

Overall, the study highlights that predicting surfactant fate in PDMPs requires considering both size exclusion and interfacial adsorption phenomena. These insights are directly relevant for designing advanced treatment under the new EU wastewater directive and for selecting membranes best suited for the removal of cationic surfactants. In real wastewater, coexisting ions, natural organic matter, and other dissolved organics may modify BAC aggregation, competitive adsorption, concentration polarization, and membrane fouling. Therefore, the quantitative retention and permeability trends reported here should be interpreted as a mechanistic baseline, while future studies should extend the analysis to mixed-matrix systems and real wastewater samples. As a next step toward practical application, future studies should address multi-component feed solutions and real wastewater matrices containing BAC together with inorganic salts and natural organic matter.

## Figures and Tables

**Table 1:** Coefficients of determination (R^2^) obtained for the linearized Hermia models fitted to experimental flux decline data.

BAC Concentration (x CMC)	Complete Blocking (*n* = 2), R^2^	Standard Blocking (*n* = 1.5), R^2^	Intermediate Blocking (*n* = 1), R^2^	Cake Filtration (*n* = 0), R^2^
X201 (RO)				
0.1	0.93	0.95	0.97	0.99
0.25	0.78	0.83	0.88	0.94
0.5	0.75	0.79	0.82	0.86
0.75	0.60	0.63	0.66	0.71
1	0.53	0.57	0.62	0.70
UTC-82V (RO)				
0.1	0.93	0.94	0.96	0.97
0.25	0.84	0.89	0.92	0.97
0.5	0.94	0.95	0.95	0.97
0.75	0.72	0.74	0.75	0.78
1	0.58	0.58	0.59	0.60
NFX (tight NF)				
0.1	0.82	0.83	0.84	0.84
0.25	0.92	0.93	0.95	0.97
0.5	0.79	0.80	0.82	0.84
0.75	0.76	0.78	0.81	0.84
1	0.55	0.56	0.56	0.57
SF10 (NF)				
0.1	0.03	0.03	0.03	0.02
0.25	0.94	0.94	0.95	0.95
0.5	0.24	0.24	0.24	0.25
0.75	0.58	0.59	0.59	0.59
1	0.68	0.68	0.69	0.70
NP030 (NF/UF)				
0.1	0.01	0.00	0.00	0.00
0.25	0.55	0.55	0.55	0.54
0.5	0.91	0.91	0.91	0.90
0.75	0.37	0.38	0.40	0.42
1	0.80	0.82	0.83	0.85

Note: Fits with low R^2^ values should be treated as indicative only, particularly in cases of negligible flux decline where discrimination between Hermia models was weak.

**Table 2:** Membrane characteristics.

	X201 (RO)	UTC-82V (RO)	NFX (NF)	SF10 (NF)	NP030 (NF/UF)
Manufacturer	TriSep	Toray	Synder	Osmonics	Microdyn-Nadir
Feed	Industrial & wastewater	Seawater	Food & industrial	Municipal & industrial wastewater	Chemical & industrial wastewater
Flux (LMH) *	60.3	24.5	57.5	53.4	16.4
MWCO (Da)	N/A	N/A	~150–300	300–500	~500–600
Rejection (%)	99.5% NaCl	99.7% NaCl	40% NaCl	≥85% NaCl	80–95%Na_2_SO_4_
Polymer	Polyamide-urea-TFC	Polyamide-TFC	Polyamide-TFC	Cellulose acetate	Polyetherosulfone
pH range (25 °C)	2–11	2–11	3–10.5	2–8	0–14

* determined by the authors (TMP = 20 bar, 22 °C).

**Table 3:** Membrane fouling models according to Hermia.

Model	Mechanism	Linearized Equation *	Schematic Diagram
Complete blocking (*n* = 2)	Each particle blocks a pore entrance; no overlap	$\ln J=\ln J_{o}+k_{cb}t$	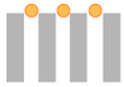
Standard blocking (*n* = 1.5)	Particles deposit inside pores, reducing effective radius	$\frac{1}{\sqrt{J}}=\frac{1}{\sqrt{J}}+k_{sb}t$	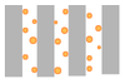
Intermediate blocking (*n* = 1)	Particles block pores randomly, but can also deposit on already blocked sites	$\frac{1}{J}=\frac{1}{J_{o}}+k_{ib}\;At$	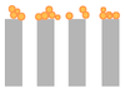
Cake filtration (*n* = 0)	Particles accumulate on membrane surface, forming a cake layer	$\frac{1}{J^{2}}=\frac{1}{J_{o}^{2}}+k_{cf}t$	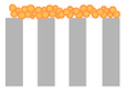

* where *J* is the permeate flux at time t, *J_o_* is the initial flux at time t = 0 min (L/m^2^h), *A* is membrane area (m^2^) and *k* is fouling parameter of each fouling mechanism.

## Data Availability

Data is contained within the article or Supplementary Material.

## Supplementary Materials

The following supporting information can be downloaded at: https://www.mdpi.com/article/10.3390/molecules31091532/s1, Figure S1: Normalized flux (*J*/*J*_0_) during 120-min filtration for the membranes at different BAC concentrations (TMP = 20 bar, T = 22 °C).
