# Supplementary material for: Fate of Benzalkonium Chloride in Nanofiltration and Reverse Osmosis: Mechanisms of Retention and Membrane Response
Source: Molecules. 2026 May 5;31(9):1532. doi: 10.3390/molecules31091532 (PMC13165109; doi:10.3390/molecules31091532)
Supplement: Supplementary file 1 [file molecules-31-01532-s001.zip › molecules-4234371-supplementary.pdf]

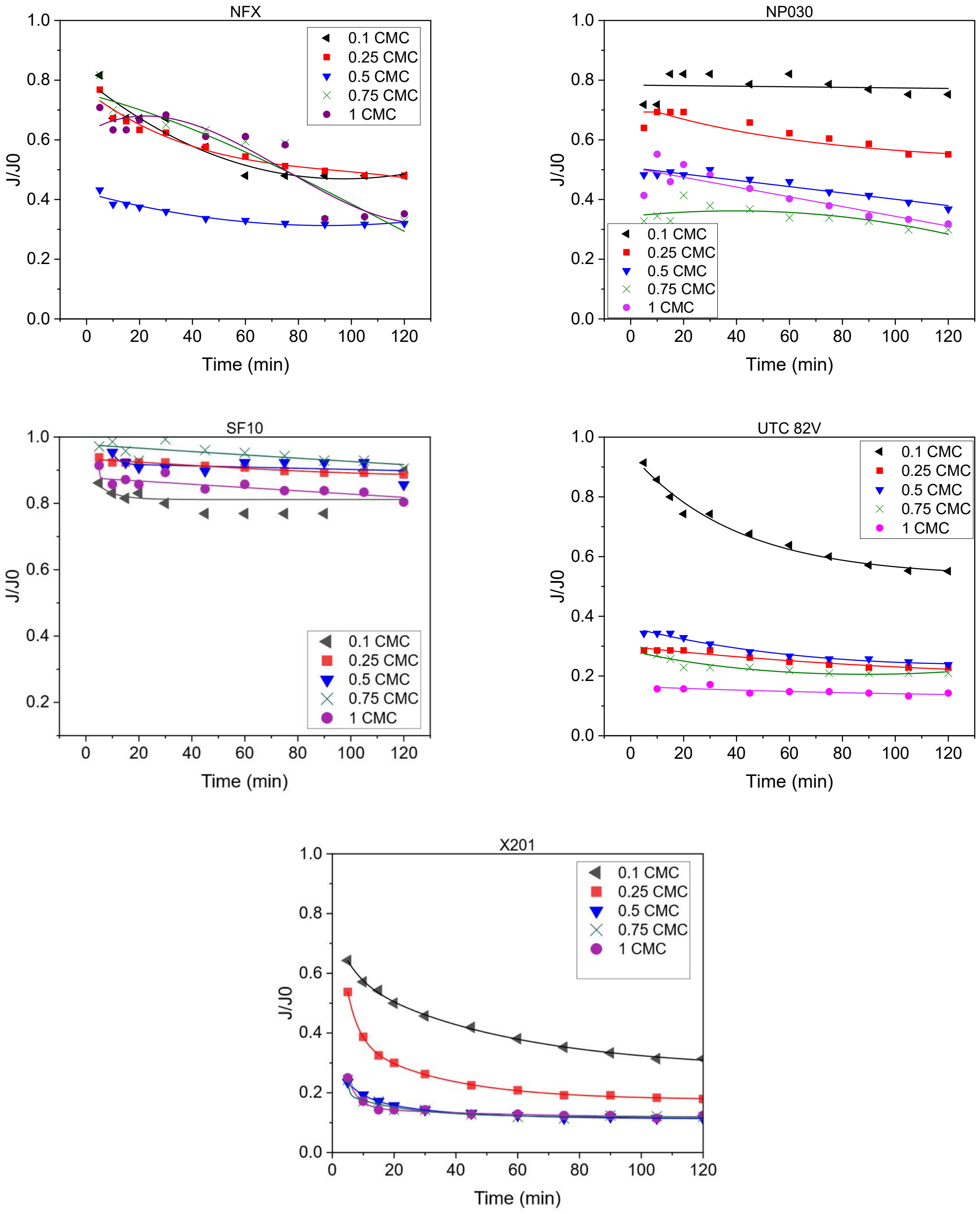

Fig. S1. Normalized flux (J/J_0) during 120-minute filtration for the membranes at different BAC concentrations (TMP = 20 bar, T = 22 °C).
